# The Performance of Nine Commercial Serological Screening Assays for the Diagnosis of Lyme Borreliosis: a Multicenter Modified Two-Gate Design Study

**DOI:** 10.1128/spectrum.00510-22

**Published:** 2022-03-17

**Authors:** B. J. A. Hoeve-Bakker, Mark Jonker, Afke H. Brandenburg, P. Martijn den Reijer, Foekje F. Stelma, Alje P. van Dam, Tamara van Gorkom, Karen Kerkhof, Steven F.T. Thijsen, Kristin Kremer

**Affiliations:** a Centre for Infectious Disease Control, National Institute for Public Health and the Environment (RIVM), Bilthoven, the Netherlands; b Department of Medical Microbiology and Immunology, Diakonessenhuis Hospital, Utrecht, the Netherlands; c Izore Centre for Infectious Diseases Friesland, Leeuwarden, the Netherlands; d Department of Medical Microbiology and Infection Prevention, Gelre Hospitals Apeldoorn, Apeldoorn, the Netherlands; e Radboud University Nijmegen Medical Centre, Nijmegen, the Netherlands; f Department of Medical Microbiology, Amsterdam University Medical Center, Amsterdam, the Netherlands; University of North Dakota

**Keywords:** *Borrelia*, CLIA, ELISA, IgG, IgM, Lyme borreliosis, Lyme disease, evaluation, performance, serology

## Abstract

In this retrospective study, the performance of nine serological screening assays for Lyme borreliosis (LB) diagnostics was evaluated using a study population of LB cases and controls. Sera derived from 74 well-defined LB cases and 122 controls were included. The LB cases were diagnosed with erythema migrans (EM; *n* = 11), Lyme neuroborreliosis (LNB; *n* = 35), Lyme arthritis (LA; *n* = 20), or acrodermatitis chronica atrophicans (ACA; *n* = 8). Controls comprised 74 age- and gender-matched healthy individuals and 48 patients with other diseases with anticipated high rates of cross-reactivity. The assays under evaluation were selected based on a literature review and expected continued availability with CE marking under the new *in vitro* diagnostic regulation (European Union) 2017/746. The overall sensitivity (IgG and IgM results combined) among LB cases ranged between 54.5% (6 of 11) and 90.9% (10 of 11) for EM patients and between 97.1% (34 of 35) and 100% for patients with LNB, LA, and ACA. The positivity rate ranged between 8.1% (6 of 74) and 29.7% (22 of 74) among the healthy controls and between 22.9% (11 of 48) and 64.6% (31 of 48) among the cross-reactivity controls. The IgM results were more heterogeneous than the IgG and IgM/IgG results and did not contribute to the overall sensitivity but substantially increased the positivity rates among the controls. In conclusion, all evaluated *Borrelia* serological screening assays performed comparably with respect to early- and late-disseminated LB. The addition of an IgM assay to the screening of *Borrelia*-specific IgG antibodies had no added value for the diagnosis of Lyme borreliosis.

**IMPORTANCE** Serology plays an important role in the diagnosis of Lyme borreliosis. Guidelines prescribe a two-tier testing algorithm in which a highly sensitive screening assay is used for screening and reactive sera are retested with an immunoblot to reduce false positivity rates. Recently, two commonly used screening assays were discontinued, including the very well-performing C6 Lyme enzyme-linked immunosorbent assay (ELISA) (Immunetics). This study provides an evaluation of the performance of nine different *Borrelia* serology screening assays, eight with expected future availably and the C6 Lyme ELISA, using a well-defined study panel of Lyme borreliosis patients, healthy population controls, and cross-reactivity controls. Evaluation data on multiple assays aid diagnostic laboratories in their choice for a reliable *Borrelia* serology screening assay to improve their diagnostic algorithm for Lyme borreliosis.

## INTRODUCTION

Lyme borreliosis (LB) is caused by spirochetes of the Borrelia burgdorferi sensu lato complex, which are transmitted by infected ticks of the genus Ixodes. In the Netherlands, 1.5 million tick bites are reported annually, resulting in approximately 27,000 LB infections (1) and substantial disease burden (2).

The diagnosis of LB is based on clinical symptoms and should be supported by laboratory tests, except for erythema migrans (EM), which is a clinical diagnosis. As the sensitivity of spirochete detection methods, such as culture and PCR, vary considerably (range, less than 10 to 83%) (3), the diagnosis of LB is often supported by the detection of Borrelia-specific antibodies. The sensitivity of enzyme immunoassays (EIAs) increases with disease duration, ranging from 50% for EM to almost 100% for late disease manifestations (4). The average specificity of these assays is 95%. To increase specificity, guidelines recommend a two-tier testing strategy in which a highly sensitive EIA is used for screening, and reactive sera are retested using an immunoblot (IB) assay (5).

Over the years, many studies have compared various Borrelia serological assays on diverse sample populations (6–11). In some of these studies, LB patients were ill-defined, and in many of these studies, assays were compared that are no longer commercially available. For instance, the production of two frequently used enzyme-linked immunosorbent assays (ELISAs) for the detection of Borrelia-specific antibodies, the C6 Lyme ELISA (Immunetics, Boston, MA) and the Enzygnost Lyme Link VlsE ELISA (Siemens Healthcare Diagnostics GmbH, Marburg, Germany), has recently been discontinued. In the Netherlands, 22 of 52 laboratories that participated in the 2019 Borrelia proficiency testing program organized by the Dutch Foundation for Quality Assessment in Medical Laboratories (SKML) used at least one of these ELISAs (A.H. Brandenburg, personal communication). Consequently, these laboratories had to implement a different Borrelia serological screening assay.

The implementation of any diagnostic assay requires both insight in how the assay compares to other currently available diagnostic assays and a thorough validation. To aid this process, this retrospective study compared eight currently available Borrelia serological screening assays and the discontinued C6 Lyme ELISA, using a well-defined study population of LB cases and controls.

## RESULTS

### Characteristics of the study population.

The demographic characteristics of the study populations are summarized in Table 1. The median age and percentage of males for LB cases and healthy controls were identical (52.5 years [range 5, to 81 years], and 63.3% males). In the cross-reactivity control group, the median age was 44.5 years (range, 13 to 83 years), and 50% were male.

**TABLE 1:** Description of the study population at inclusion of the study^*a*^

Characteristics	All LB cases (*n* = 74)	EM (*n* = 11)	LNB^*b*^		LA (*n* = 20)	ACA (*n* = 8)	Healthy controls (*n* = 74)	Cross-reactivity controls (*n* = 48)
			Definite (*n* = 27)	Possible (*n* = 8)				
Age (median [range])	52.5 (5 to 81)	58.0 (18 to 77)	41.0 (6 to 80)	50.0 (11 to 75)	52.5 (5 to 81)	65.5 (53 to 77)	52.5 (5 to 81)	44.5 (13 to 83)
Gender (no of males [%])	50 (63.3)	6 (54.5)	18 (66.7)	4 (50.0)	19 (95.0)	3 (37.5)	50 (63.3)	24 (50.0)
Phlebotomy prior to antibiotic treatment (*n* [%])	62 (83.8)	11 (100)	21 (77.8)	6 (75.0)	16 (80.0)	8 (100)	ND	ND
Positive *Borrelia* serology (*n* [%])	32 (82.1)	4 (36.4)	ND	ND	20 (100)	8 (100)	ND	ND
*Borrelia*-specific intrathecal antibody production (*n* [%])	ND	ND	26 (96.3)	4 (50.0)	ND	ND	ND	ND
*Borrelia* spp. PCR (*n* positive/*n* tested [%])	32/45 (71.1)	6/7 (85.7)	4/13 (30.8)	0/3 (0)	15/15 (100)	7/7 (100)	ND	ND

*^a^* The table shows the demographic characteristics, known serology, and PCR results of all LB cases, the four LB manifestations, and the two control groups. LB, Lyme borreliosis; EM, erythema migrans; LNB, Lyme neuroborreliosis; LA, Lyme arthritis; ACA, acrodermatitis chronica atrophicans; ND, not determined.

b As determined by the European Federation of the Neurological Societies (EFNS) guidelines (26).

The laboratory results of the LB cases provided at inclusion of the study are summarized in Table 1. Four (36.4%) of 11 EM cases were seropositive. Borrelia-specific DNA was detected in 6 (85.7%) of 7 EM cases, for whom PCR on skin biopsy was performed, including in 3 seronegative EM cases. Of the 35 Lyme neuroborreliosis (LNB) cases, 27 (77.1%) were definite LNB (dLNB) cases, and 8 (22.9%) were possible LNB (pLNB) cases. Intrathecally produced Borrelia-specific antibodies were detected in 26 of the 27 dLNB cases. For 4 of the 13 dLNB cases for whom a Borrelia PCR on cerebrospinal fluid (CSF) was done, the result was positive, including the sample without proven intrathecal Borrelia-specific antibody synthesis. Four (50.0%) of eight pLNB cases had pleocytosis, and four (50.0%) had intrathecal Borrelia-specific antibody synthesis (Table 1). None of the three pLNB cases for whom a Borrelia spp. PCR on CSF was performed had positive results. All of the Lyme arthritis (LA) cases had a positive serology result. For 15 (75.0%) of the 20 LA cases, a Borrelia-specific PCR on synovial fluid from the affected joint was performed, and all of them had a positive result. All eight acrodermatitis chronica atrophicans (ACA) patients had typical skin lesions and a positive serology result. For seven (87.5%) of eight ACA patients, Borrelia PCR on a skin biopsy sample was done, and the results were all positive.

### Sensitivity and positivity rates.

Overall, the sensitivity of the assays differed depending on the Ig class and disease manifestation. The sensitivity of the assays among LB cases ranged between 91.9 and 98.6% for overall Ig (Table 2). For assays with separate Ig measurements, the sensitivity among LB cases ranged between 45.9 and 77.0% for IgM and between 86.5 and 95.9% for IgG.

**TABLE 2:** Sensitivity and positivity rates of *Borrelia* serological screening assays^*a*^

Assay	Sensitivity (%)					Positivity rate (%)	
	All LB cases (*n* = 74)	EM (*n* = 11)	LNB (*n* = 35)	LA (*n* = 20)	ACA (*n* = 8)	Healthy controls (*n* = 74)	Cross-reactivity controls (*n* = 48)
IgM assays							
DRG IgM	45.9	36.4	68.6	25.0	12.5	10.8	35.4
Euroimmun IgM	55.4	18.2	68.6	55.0	50.0	6.8	37.5
Liaison IgM	51.4	36.4	68.6	35.0	37.5	8.1	33.3
NovaLisa IgM	55.4	45.5	77.1	30.0	37.5	23.0	41.7
Serion IgM	77.0	54.5	91.4	65.0	75.0	21.6	60.4
VirClia IgM	45.9	18.2	71.4	30.0	12.5	8.1	29.2
IgG assays							
DRG IgG	91.9	54.5	97.1	100	100	9.5	14.6
Euroimmun IgG	95.9	72.7	100	100	100	13.5	22.9
Liaison IgG	93.2	54.5	100	100	100	4.1	14.6
NovaLisa IgG	95.9	81.8	97.1	100	100	10.8	22.9
Serion IgG	86.5	63.6	82.9	100	100	8.1	16.7
VirClia IgG	95.9	72.7	100	100	100	13.5	14.6
Overall Ig results (proportion of solitary IgM results)							
DRG^*b*^	91.9 (0.0)	54.5 (0.0)	97.1 (0.0)	100 (0.0)	100 (0.0)	20.3 (10.8)	50.0 (35.4)
Euroimmun^*b*^	95.9 (0.0)	72.7 (0.0)	100 (0.0)	100 (0.0)	100 (0.0)	18.9 (5.4)	50.0 (27.1)
Liaison^*b*^	95.9 (2.7)	72.7 (18.2)	100 (0.0)	100 (0.0)	100 (0.0)	12.2 (8.1)	43.8 (29.2)
NovaLisa^*b*^	97.3 (1.4)	90.9 (9.1)	97.1 (0.0)	100 (0.0)	100 (0.0)	29.7 (18.9)	58.3 (35.4)
Serion^*b*^	95.9 (9.5)	72.7 (9.1)	100 (17.1)	100 (0.0)	100 (0.0)	27.0 (18.9)	64.6 (47.9)
VirClia^*b*^	95.9 (0.0)	72.7 (0.0)	100 (0.0)	100 (0.0)	100 (0.0)	21.6 (8.1)	39.6 (25.0)
C6 IgM/IgG^*c*^	98.6	90.9	100	100	100	10.8	25.0
Euroimmun IgM/IgG^*c*^	97.3	81.8	100	100	100	8.1	22.9
Zeus IgM/IgG^*c*^	94.6	63.6	100	100	100	9.5	29.2

*^a^*The table shows the sensitivity and positivity rates of the nine *Borrelia* serological screening assays among all LB cases, the four LB manifestations, and the two control groups included in this study. For the calculation of the sensitivities and positivity rates, equivocal results were considered positive. ACA, acrodermatitis chronica atrophicans; EM, erythema migrans; LA, Lyme arthritis; LB, Lyme borreliosis; LNB, Lyme neuroborreliosis.

*^b^*For assays with separate IgM and IgG measurements, the overall Ig assay positivity was based on the combined IgM and IgG results (i.e., negative when both IgM and IgG were negative, and positive when at least one of these was positive).

*^c^*The proportion of solitary IgM results is not applicable for assays with combined IgM and IgG measurements.

The overall Ig sensitivity for most assays to detect systemic LB infections was excellent, which was mainly attributable to the high sensitivities of the IgG assays (Table 2). LNB, LA, and ACA were detected by all assays (100% sensitivity), except for the NovaLisa and DRG assays, which missed one and the same LNB patient (97.1% sensitivity). The sensitivity of the IgM assays varied by disease manifestation and ranged from 68.6 to 91.4% for LNB, from 25.0 to 65.0% for LA, and from 12.5 to 75.0% for ACA. The IgM result did not contribute to the overall Ig sensitivity for most of the assays, except for the Serion ELISA, for which a solitary IgM reactivity of 18.1% was found.

Overall Ig sensitivity of the assays among EM cases was more variable, with sensitivities ranging from 54.5 to 90.9%. The sensitivities of assays measuring IgM and IgG separately among EM cases ranged from 18.2 to 54.5% and from 54.5 to 81.8%, respectively. A solitary IgM response among EM cases was reported for the NovaLisa and Serion assays (both 9.1%) and the Liaison assay (18.2%).

The positivity rates of the assays varied among both healthy and cross-reactivity controls (Table 2). Among the 74 healthy controls, positivity rates ranged from 6.8 to 23.0% for IgM, from 4.1 to 13.5% for IgG, and from 8.1 to 29.7% for overall Ig. Solitary IgM results were obtained for all assays, and their contribution to the overall Ig positivity rate ranged from 5.4 to 18.9%.

The positivity rates within the cross-reactivity control group varied the most and ranged from 29.2 to 60.4% for IgM, from 14.6 to 22.9% for IgG, and from 25.5 to 64.6% for overall Ig (Table 2). The contribution of a solitary IgM response to the overall Ig results ranged from 25.0 to 47.9%. The results for each disease included in the cross-reactivity control group are summarized in the supplemental material (Table S1).

### Receiver operating characteristic (ROC) curve analysis.

**FIG 1:**
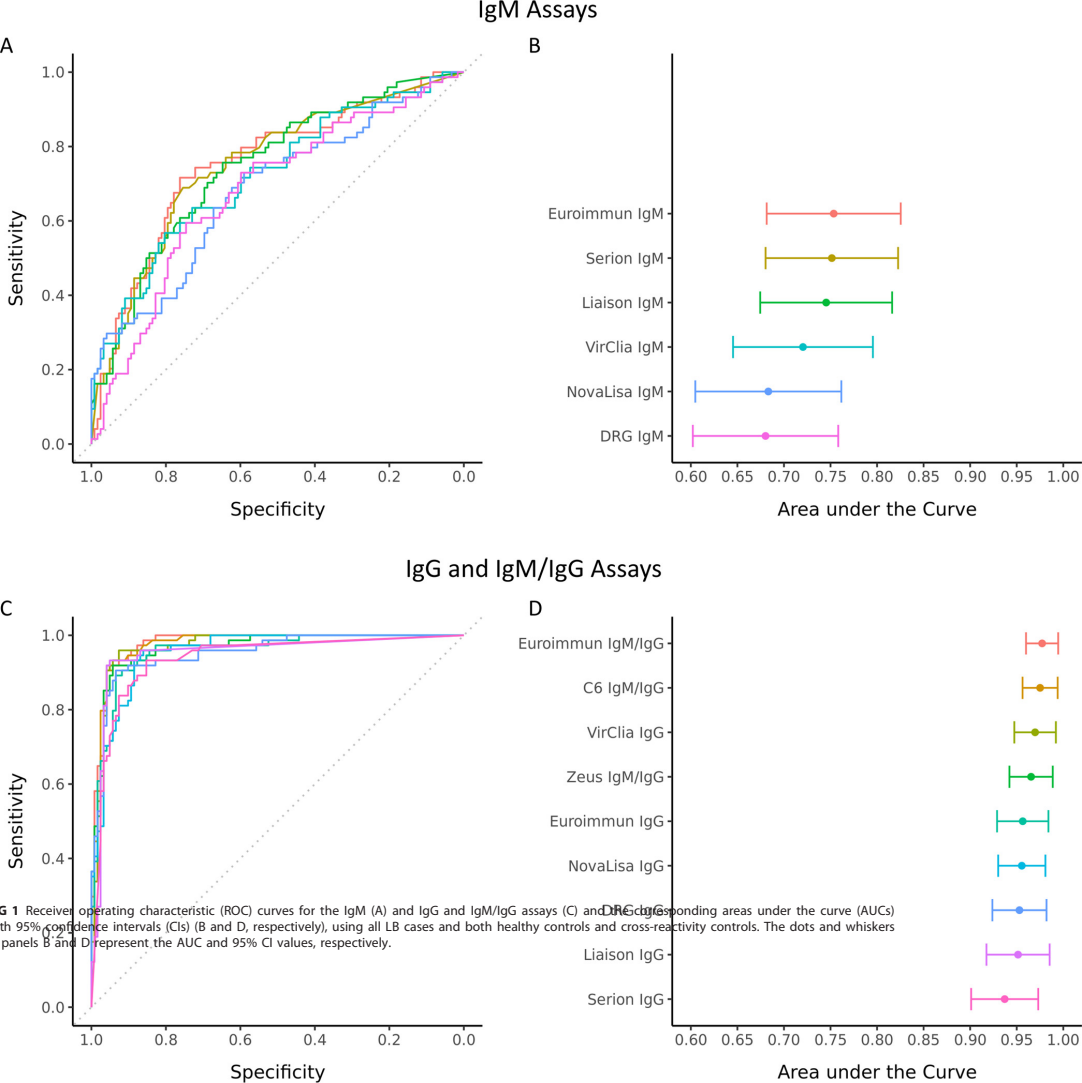
Receiver operating characteristic (ROC) curves for the IgM (A) and IgG and IgM/IgG assays (C) and the corresponding areas under the curve (AUCs) with 95% confidence intervals (CIs) (B and D, respectively), using all LB cases and both healthy controls and cross-reactivity controls. The dots and whiskers in panels B and D represent the AUC and 95% CI values, respectively.

ROC curve analysis was performed for all assays (Fig. 1). For the IgM assays, the AUCs ranged from 0.680 to 0.753 (Fig. 1B). Overlapping 95% confidence intervals (CIs) indicated that no significant differences were observed between areas under the curve (AUCs) (Fig. 1B). The ROC curves of the combined IgM/IgG assays were comparable to those measuring IgG only (Fig. 1C). The AUCs ranged from 0.965 to 0.977 for combined IgM/IgG assays and from 0.937 to 0.970 for IgG assays and were not significantly different (Fig. 1D).

The ROC curves of the overall Ig results were determined using logistic regression modeling. For most assays, model 2 (the independent model) was most optimal, except for the DRG and VirClia assays, for which model 3 performed best (data not shown). Irrespective of the assay, the AUC based on the overall Ig results was lower than for IgG only, although this was not significant (data not shown).

### Inter-assay agreement.

**FIG 2:**
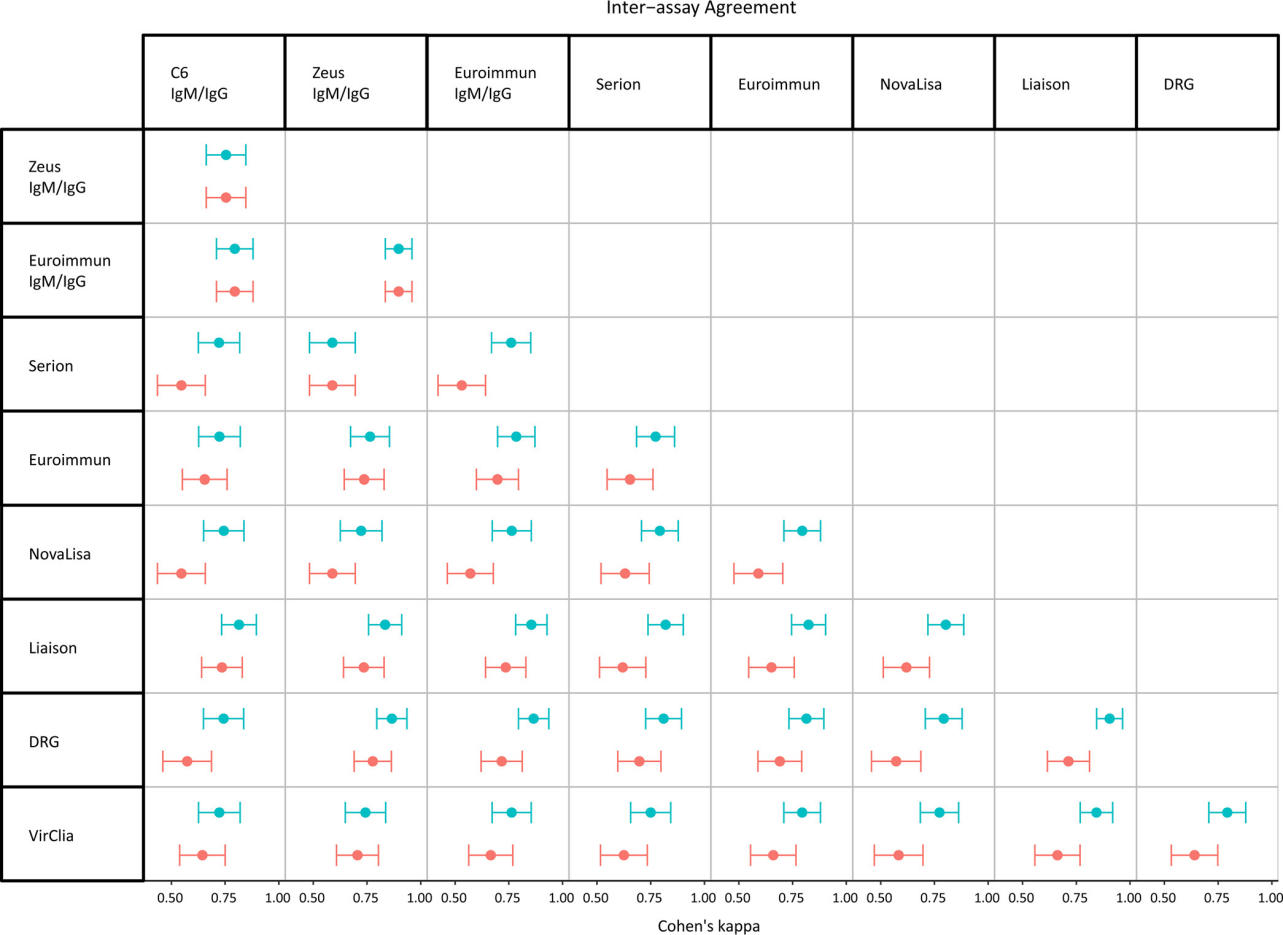
A forest plot of the inter-assay agreement between any two assays for IgG results (blue) and overall Ig results (red) showing Cohen’s ĸ values (dots) with 95% confidence intervals (whiskers).

The agreement between any two assays based on the overall Ig results was fair to excellent, with ĸ values ranging from 0.53 to 0.78. The level of agreement increased substantially for most assays when IgM results were excluded from the comparison (ĸ values of 0.59 to 0.91) as is shown in Fig. 2. Excellent agreement (ĸ > 0.75) was observed between most of the IgG and IgM/IgG assays. The lowest agreement was observed between the Zeus IgM/IgG assay and the Serion IgG assay (ĸ, 0.59; 95% CI, 0.48 to 0.70).

## DISCUSSION

This study compared the performance of nine CE-certified Borrelia serological screening assays, all of which, other than the C6 Lyme ELISA, are anticipated to be continued under the new European regulation for *in vitro* diagnostic (IVD) medical devices (CE-IVDR) (12). To meet the various demands of diagnostic laboratories, assays based on whole-cell antigens, recombinant antigens, and a combination of both were included. The assays under investigation represent a wide selection of economically accessible platforms and formats, in which the assays can be performed both manually and on automated systems and are suitable for both high and low throughput.

The overall sensitivity of the assays for clinically diagnosed LB cases was excellent (range, 91.9 to 98.6%; overall Ig results). The sensitivity among patients with a disseminated Lyme manifestation (i.e., LNB, LA, and ACA) was higher than among patients with EM and was in concordance with previous reports (4, 7, 13). A negative test result among EM patients can be explained by misdiagnosis of unclear skin manifestations, undetectable antibody levels that are still building up, or an abrogated antibody response due to prompt antibiotic treatment (3). The data on EM patients in this study support the national guideline, issued by the Institute for Healthcare Improvement (CBO) that discourages serological testing for EM patients and recommend diagnosis and treatment of EM solely based on clinical symptoms (5).

The positivity rates in the cross-reactivity control group were higher than those among healthy controls. The IgG positivity rates among healthy controls most likely indicate past LB infection as these were comparable to the estimated IgG background seropositivity rate of 4 to 8% in the Netherlands (5) and with seroprevalence rates reported in Western European countries (14, 15). In contrast, IgM positivity rates were much higher and might be explained by cross-reactivity with antibodies raised against heterologous bacteria and autoimmune diseases or by polyclonal B-cell activation (16). The higher positivity rates among the cross-reactivity controls confirmed what has been published before (6, 7, 11, 17) and underlined that positive IgM serology results should be interpreted with care, especially when clinical symptoms are unclear.

The overall Ig positivity rates in the healthy control group ranging from 8.1 to 29.7% are undesirable and underscore the need for pretest probability estimation as well as a two-tier testing strategy for clinical diagnosis of LB. False-positive test results have a negative impact on positive predictive values (PPVs), and the PPV increases with an increasing pretest probability (13). Therefore, the pretest probability should be estimated prior to serologic testing based on exposure and clinical symptoms of the individual patient, as well as disease prevalence (5, 13). Additionally, retesting reactive sera with a second-tier assay will reduce false-positivity rates (5, 13).

The results in this study show that all assays measuring IgG and IgM/IgG performed comparably, with high AUC values that reflect high sensitivities, at least for LB patients with a disseminated Lyme manifestation. The degree of homogeneity in assay performance was also reflected by the high inter-assay agreement and the narrow 95% CIs of the AUCs. These results are in line with another recent study (6) and suggest that the assays measuring IgG and IgM/IgG in this study are equally suitable for use in clinical practice.

In contrast to the IgG and IgM/IgG assays, the IgM assays performed more heterogeneously as shown by wider 95% CIs of the respective AUCs, although the differences in AUCs between the IgM assays were not statistically significant. However, the ROC curves of the IgM assays had significantly lower AUCs than those of the IgG and IgM/IgG assays. These differences in assay performance based on antibody isotype are also observed for other infectious diseases, such as chronic pulmonary aspergillosis (18) and chikungunya virus (19).

In clinical practice, serological testing for Borrelia-specific antibodies often encompasses both IgM and IgG testing. This study showed that the proportion of solitary IgM responses was higher among both control groups than among LB cases. Moreover, combining IgM and IgG results in fact decreased the inter-assay agreement substantially for most manufacturers. Overall, the results of this study suggest that the use of IgM assays for screening for Borrelia-specific antibodies may result in overdiagnosis and mistreatment. This phenomenon was elaborately discussed (13) and evaluated in clinical practice (20). Therefore, testing the IgM response only when clinical symptoms point toward early infection seems more appropriate. Even then, solitary IgM reactivity should be interpreted carefully because false positivity due to cross-reactivity or past infection should not be excluded as IgM antibodies can be detected for months to years after active LB (21).

With tightened European regulations regarding the CE-marking of IVDs being effectuated in 2022, continuation of some diagnostic assays may no longer be feasible for manufacturers (22). One of the strengths of this study is that eight assays were compared that are currently available for the detection of Borrelia-specific antibodies in diagnostic laboratories and have expected continued availability under the new CE-IVDR. Moreover, this study contributed to the intra-laboratory validation of Borrelia screening assays, which is a requirement for ISO 15189 accreditation.

For most LB manifestations, the detection of Borrelia-specific antibodies is an important part of the diagnosis. Although clinically well-defined, the LB cases included in this study were partly selected based on positive serology in the participating laboratories, which could have resulted in an overestimation of the sensitivity of the assays under investigation. To reduce this selection bias, the samples were obtained from laboratories that used different screening assays. Additionally, all samples were analyzed using all assays, allowing assessment of the inter-assay agreement and identifying cases that were missed by one or more assays.

Two-gate design studies including cases and controls may suffer from spectrum effects (i.e., various accuracy measures across different study populations) and limited-challenge bias (i.e., including only the sickest cases), leading to overestimation of accuracy measures (4, 23). In this study, spectrum effects were reduced to a minimum by applying a modified two-gate study design that included both healthy and cross-reactivity controls; a combination of both two-gate designs described by Rutjes et al. (23). Although this study focused mainly on systemic LB infections, limited-challenge bias was reduced by inclusion of EM patients. Reverting to a prospective one-gate design, by including patients suspected of having LB, could have further improved the study setup (23). However, inclusion of sufficient numbers of all LB manifestations would have taken considerably more time.

In conclusion, all investigated assays showed excellent sensitivities, although two-tier testing is needed to reduce the positivity rates. The diagnosis of LB is best supported by using an IgG or IgM/IgG assay, and most assays measuring IgM did not contribute to the diagnosis of LB. Because the presence of IgM antibodies is not proof of an active infection, the addition of an IgM assay to the Borrelia screening of IgG antibodies appears unfavorable. If reported to clinicians at all, the IgM results should be interpreted with care.

## MATERIALS AND METHODS

### Study design.

A panel of 196 sera was assembled using a modified two-gate study design (23) in which cases and controls are typically selected from different populations and used to estimate the sensitivity and specificity, respectively. The panel consisted of LB cases selected from a population of LB patients, healthy controls selected from the general Dutch population, and patients diagnosed with possibly cross-reacting diseases (cross-reactivity controls).

The Borrelia serological screening assays were selected based on performance demonstrated in earlier studies and the type of antigens used. Furthermore, only those assays were chosen for which a continued CE marking under the new CE-IVDR (12) is expected, based on the respective manufacturer’s statements. In total, eight assays were included, and the results were compared with the discontinued C6 Lyme ELISA. The results obtained in this study were reported in adherence to the Standards for Reporting Diagnostic Accuracy (24).

### Study population.

Seventy-four LB cases were selected to reflect the most prevalent LB manifestations in the Netherlands, as described by Hofhuis et al. (25), with an emphasis on systemic Lyme infections in which serology plays a role in the diagnosis. These LB cases included 11 physician-diagnosed patients with EM, 35 LNB patients, 20 LA patients, and 8 ACA patients (Table 1).

All LB cases were defined according to the CBO guideline Lyme Disease (5). The LNB patients were defined according to the guidelines of the European Federation of the Neurological Societies (EFNS) (26). A LNB patient was classified as a dLNB patient when the following three criteria were met (i) neurological symptoms in the absence of other possible causes; (ii) CSF pleocytosis; and (iii) intrathecally produced Borrelia-specific antibodies or detection of borrelial DNA in CSF. A LNB patient was classified as a pLNB patient when the first criterion was met and either the second or the third criterion was fulfilled.

The sera were obtained from five laboratories in the Netherlands and were eligible for inclusion if at least 500 μl of serum was available to perform all assays under investigation. For all LB cases, serology and/or Borrelia PCR was performed at the laboratory that provided the samples. For EM, LA, and ACA cases, previous serology consisted of a two-tier test strategy by using either the C6 Lyme ELISA or the Liaison Borrelia IgG (DiaSorin S.p.A, Saluggia, Italy) assay, followed by IB confirmation by recomLine IgG (Mikrogen, Neuried, Germany) or anti-Borrelia (IgG) EUROLINE-RN-AT (Euroimmun AG, Lübeck, Germany). For LNB cases, the IDEIA Lyme neuroborreliosis kit (Oxoid, Hampshire, UK) and a Borrelia-specific antibody index calculation were used to determine intrathecal Borrelia-specific antibody synthesis. For a subset of LB cases, the results of a Borrelia-specific in-house PCR were also available.

Healthy controls (*n* = 74) were selected from the third cross-sectional population-based seroprevalence study carried out in the Netherlands in 2016 and 2017 (PIENTER-3) (27) and were age- and gender-matched to the LB cases to correct for possible selection bias. The selected cross-reactivity controls (*n* = 48) comprised patients with positive serology for an infectious disease (Epstein-Barr virus [*n* = 10], cytomegalovirus [*n* = 9], leptospirosis [*n* = 8], syphilis [*n* = 7], hepatitis C virus [acute *n* = 2, chronic *n* = 2], and Helicobacter pylori [*n* = 3]) or patients with autoimmune disease (tested positive for anti-nuclear antibodies [*n* = 4] or rheumatoid factor [*n* = 3]). For both control groups, no history of LB was reported.

### Sample handling and storage.

All serum samples were assigned a study code to ensure anonymous handling. Approval of the local ethics committee was not necessary, since the main purpose of this study was to evaluate assay performance, for which leftover material was used.

Except for shipment to the National Institute for Public Health and the Environment at room temperature, the samples were stored and kept at −20°C until testing. Before testing, the samples were thawed to room temperature, homogenized, and centrifuged.

### *Borrelia* serological screening assays.

An overview of the assays included in this study is presented in Table 3. The C6 IgM/IgG, the Zeus IgM/IgG, and the Euroimmun IgM/IgG assays measure IgG and IgM simultaneously, whereas the Euroimmun, Liaison, NovaLisa, Serion, DRG, and VirClia assays measure IgG and IgM separately. All assays were performed according to the respective manufacturer’s instructions. The C6 IgM/IgG was performed on a Dynex DS2 automated ELISA instrument (Dynex DS2, Dynex Technologies, Chantilly, VA) or a Serion Immunomat ELISA robot instrument (Institut Virion\Serion GmbH, Würzburg, Germany) at the laboratory of origin or performed manually at the National Institute for Public Health and the Environment (RIVM) if no C6 IgM/IgG result was reported at the time of collection. The Euroimmun, NovaLisa, and Zeus assays were performed manually at the RIVM. The Serion ELISAs were performed at the RIVM on the Serion Immunomat ELISA robot instrument. The Liaison assays were processed on the LIAISON XL Analyzer (DiaSorin S.p.A, Saluggia, Italy) at the Diakonessenhuis Utrecht. The VirClia Monotests and the DRG ELISAs were processed on the VirClia System (Vircell S.L., Granada, Spain) at Gelre Hospitals Apeldoorn. The serological test results were reported as negative, equivocal, or positive.

**TABLE 3:** Overview of the selected *Borrelia* serological screening assays, types of antigens, and methods used^*a*^

Abbreviation	Assay (manufacturer)	Antigens^*b*^	Method
DRG IgM	*Borrelia* 14 kDa + OspC IgM (DRG Diagnostics GmbH, Marburg, Germany)	Native OspC (Ba), 14-kDa flagellin fragment (Bsl)	ELISA
Euroimmun IgM	Anti-*Borrelia* ELISA (IgM) (Euroimmun AG, Lübeck, Germany)	SDS extract (Bss, Bg, Ba); enhanced OspC expression	ELISA
Liaison IgM	Liaison *Borrelia* IgM Quant (DiaSorin S.p.A, Saluggia, Italy)	OspC (Ba), VlsE (Bg)	CLIA
NovaLisa IgM	NovaLisa B. burgdorferi IgM (NovaTec Immundiagnositca GmbH, Dietzenbach, Germany)	OspC (Ba, Bg), p41i (Bg)	ELISA
Serion IgM	Serion ELISA classic B. burgdorferi IgM (Institute Virion\Serion GmbH, Würzburg, Germany)	Whole-cell lysates (Ba, Bg)	ELISA
VirClia IgM	*Borrelia* VirClia IgM Monotest (Vircell S.L., Granada, Spain)	OspC (Ba, Bg, Bss, Bsp), VlsE (Bg), p41i (Bsl), p39 (Bsl), p17 (Bsl), OspE (Bsl)	CLIA
DRG IgG	*Borrelia* IgG + VIsE (DRG Diagnostics GmbH, Marburg, Germany)	crude lysate (Bss, Ba, Bg), VlsE (Bss, Ba, Bg)	ELISA
Euroimmun IgG	Anti-*Borrelia* plus VlsE ELISA (IgG) (Euroimmun AG, Lübeck, Germany)	SDS extract (Bss, Bg, Ba), VlsE (Bss)	ELISA
Liaison IgG	Liaison *Borrelia* IgG (DiaSorin S.p.A, Saluggia, Italy)	VlsE (Bg)	CLIA
NovaLisa IgG	NovaLisa B. burgdorferi IgG (NovaTec Immundiagnositca GmbH, Dietzenbach, Germany)	OspC (Bss, Bg), p100 (Ba), p18 (Ba), p41i (Bg)	ELISA
Serion IgG	Serion ELISA classic B. burgdorferi IgG (Institute Virion\Serion GmbH, Würzburg, Germany)	Whole-cell lysates (Ba, Bg), VlsE (Bg)	ELISA
VirClia IgG	*Borrelia* VirClia IgG Monotest (Vircell S.L., Granada, Spain)	VlsE (Bsl), p41i (Bsl), p17 (Bsl)	CLIA
C6 IgM/IgG	C6 Lyme ELISA (Immunetics, Boston, MA)	Synthetic C6 peptide (derived from VlsE)	ELISA
Euroimmun IgM/IgG	Lyme ELISA (IgG/IgM) (Euroimmun AG, Lübeck, Germany)	VlsE (Bss), OspC (Bss)	ELISA
Zeus IgM/IgG	*Borrelia* VlsE1/pepC10 IgG/IgM Test System (Zeus Scientific Inc., Brachburg, NJ)	VlsE1, pepC10 (10-mer peptide at C terminus of OspC)	ELISA

*^a^* Ba, B. afzelii; Bg, B. garinii; Bsl, B. burgdorferi sensu lato; Bsp, B. spielmanii; Bss, B. burgdorferi sensu stricto; ELISA, enzyme-linked immunosorbent assay; CLIA, chemiluminescent immunoassay.

b The antigens are recombinant unless otherwise stated.

### Data analysis.

Sensitivity was defined as the proportion of LB cases having a positive test result. For each assay, the sensitivity was determined for the entire panel of LB cases and for each of the individual LB manifestations. The positivity rate for each of the two control groups was defined as the proportion of controls having a positive test result. For the calculation of the sensitivity and the positivity rates, equivocal test results were considered positive.

The results of IgM and IgG assays were analyzed both separately and combined (“overall Ig results”). Overall Ig results were considered negative when both IgM and IgG were negative and positive when at least one of these was positive.

ROC curves were constructed to assess the sensitivity and the specificity of the assays. Assay comparison was performed based on the area under the curve (AUC) values and 95% CI values calculated in R version 4.0.2 (28) using the pROC package (29). The ROC curves of the overall Ig results were determined using logistic regression modeling. For each assay measuring IgM and IgG separately, the following five models were constructed: (i) saturated model of numerical IgM and IgG results; (ii) independence model of numerical IgM and IgG results; (iii) models 1 and 2 combined; (iv) model 3 excluding IgM results as independent variable; and (v) model 3 excluding IgG results as independent variable. The optimal model (with the smallest Akaike Information Criterion [AIC]) was used for further ROC curve analyses.

Cohen’s κ and 95% CI values were computed to assess the agreement between any two assays (inter-assay agreement) using the Visualizing Categorical Data package (30). For assays measuring IgM and IgG separately, the κ values were calculated for IgG results only and for overall Ig results. Excellent (ĸ > 0.75), fair to good (ĸ, 0.40 to 0.75), and poor agreement (ĸ less than 0.40) categories were qualified according to the work of Fleiss et al. (31).
