# Supplementary material for: The Performance of Nine Commercial Serological Screening Assays for the Diagnosis of Lyme Borreliosis: a Multicenter Modified Two-Gate Design Study
Source: Microbiol Spectr. 2022 Mar 17;10(2):e00510-22. doi: 10.1128/spectrum.00510-22 (PMC9045392; doi:10.1128/spectrum.00510-22)
Supplement: SUPPLEMENTAL FILE 1 — Supplemental material. Download SPECTRUM00510-22_Supp_1_seq5.pdf, PDF file, 0.1 MB [file spectrum00510-22_supp_1_seq5.pdf]

Supplemental material

Supplemental Table S1: The number of positive results among the cross-reactivity controls in the nine *Borrelia* screening assays shown for each disease separately.

	Positive result[1] (n)							
	**EBV** n=10	**CMV** n=9	**Lepto-spira** n=8	**Syphilis** n=7	**HCV** n=4	***Helico-bacter pylori*** n=3	**ANA** n=4	**RF** n=3
**IgM assays**								
**DRG IgM**	6	5	4	2	0	0	0	0
**Euroimmun IgM**	4	7	1	3	1	0	2	0
**Liaison IgM**	5	5	3	2	0	0	1	0
**NovaLisa IgM**	7	4	3	2	1	0	2	1
**Serion IgM**	7	8	7	4	1	0	2	0
**VirClia IgM**	4	4	2	1	1	0	1	1
**IgG assays**								
**DRG IgG**	0	2	0	3	1	1	0	0
**Euroimmun IgG**	0	2	1	5	1	2	0	0
**Liaison IgG**	0	1	0	4	1	0	0	1
**NovaLisa IgG**	1	1	0	4	2	2	1	0
**Serion IgG**	1	1	0	3	1	2	0	0
**VirClia IgG**	0	1	1	2	2	1	0	0
**Overall Ig result (solitary IgM result)**								
**DRG[2]**	6 (6)	7 (5)	4 (4)	5 (2)	1 (0)	1 (0)	0 (0)	0 (0)
**Euroimmun[2]**	4 (4)	8 (6)	1 (0)	6 (1)	1 (0)	2 (0)	2 (2)	0 (0)
**Liaison[2]**	5 (5)	6 (5)	3 (3)	4 (0)	1 (0)	0 (0)	1 (1)	1 (0)
**NovaLisa[2]**	8 (7)	5 (4)	3 (3)	4 (0)	2 (0)	2 (0)	3 (2)	1 (1)
**Serion[2]**	7 (6)	8 (7)	7 (7)	4 (1)	1 (0)	2 (0)	2 (2)	0 (0)
**VirClia[2]**	4 (4)	5 (4)	3 (2)	2 (0)	2 (0)	1 (0)	1 (1)	1 (1)
**C6 IgM/IgG[3]**	2	4	0	3	1	0	1	1
**Euroimmun IgM/IgG[3]**	1	5	0	3	1	0	1	0
**Zeus IgM/IgG[3]**	3	6	0	3	1	1	0	0

EBV: Epstein-Barr virus, CMV: cytomegalovirus, ANA: anti-nuclear antibodies, HCV: hepatitis C virus, RF: rheumatoid factor.

[1] Equivocal test results were considered positive.

[2] For the assays with separate IgM and IgG measurement, the separate IgG and IgM results were combined to determine the overall Ig positivity and was negative when both IgM and IgG were negative, and positive when at least one of those was positive.

[3] The proportion of solitary IgM result is not applicable for assays with combined IgM and IgG measurement.
